# Supplementary material for: Humans and neural networks show similar patterns of transfer and interference during continual learning
Source: Nat Hum Behav. 2025 Oct 30;10(1):111–25. doi: 10.1038/s41562-025-02318-y (PMC12846915; doi:10.1038/s41562-025-02318-y)
Supplement: Supplementary file 1 — Supplementary Figs. 1–18 and text. [file 41562_2025_2318_MOESM1_ESM.pdf]

nature human behaviour

Supplementary information https://doi.org/10.1038/s41562-025-02318-y

Humans and neural networks show similar patterns of transfer and interference during continual learning

In the format provided by the authors and unedited

Contents

1 Task Design **2**
1.1 Participant instructions . . . 2
1.1.1 Introductory instructions . . . 2
1.1.2 Re-test instructions . . . 2
1.1.3 Debrief instructions . . . 2

2 Additional ANN analyses of transfer / interference **4**
2.1 ANN implementation with non-linearities (ReLU) . . . 6

3 Behavioural Effects of Task Similarity **8**

4 Model Fitting **12**
4.1 Interference Model Fitting . . . 12
4.1.1 EM Algorithm for Mixture of von Mises Distributions . . . 12
4.1.2 Interference Sanity Checks . . . 12
4.1.3 Parameter Recovery and Permutation Test Validation . . . 13
4.2 Characterizing Interference Errors: Swaps vs. Biases . . . 15
4.3 Model Variant: Fitting Interference Offset . . . 16

5 Individual Differences **19**
5.1 Accuracy Across Training in Lumpers, Splitters, Rich, and Lazy Networks . . . 19
5.2 Excluding Random Responders . . . 21

6 Mitigating Interference in ANNs **22**
6.1 Mitigating Interference: EWC, Replay, and Modular Architectures . . . 23
6.2 EWC . . . 23
6.3 Replay . . . 23
6.4 Modular Architecture . . . 23
6.5 Comparing human profiles to model behaviours. . . . 25

List of Figures

S1 Matched task rules across conditions. . . . 3
S2 Evolution of ANN hidden layer subspaces. . . . 4
S3 Interference across a continuum of rule similarities. . . . 5
S4 ANN results do not depend on hidden unit size. . . . 6
S5 Patterns of transfer and interference in a two-layer ReLU network. . . . 7
S6 Main results separated by Discovery and Replication sample. . . . 8
S7 Retest accuracy. . . . 9
S8 Raw accuracy split by response type. . . . 10
S9 Simulated transfer under alternative strategies. . . . 11
S10 Participant response histograms and model fits (Far). . . . 14
S11 Participant response histograms and model fits (Near). . . . 15
S12 Swap vs. bias interference errors. . . . 17
S13 Fitted interference weight for Lumpers. . . . 18
S14 Comparing response distributions in lumpers and splitters. . . . 19
S15 Training accuracy for splitters vs. lumpers and rich vs. lazy networks. . . . 20
S16 Continuum of rich to lazy learning. . . . 22
S17 Representational solutions under different model architectures . . . 25
S18 Transfer and interference for different model architectures. . . . 26

1 Task Design

1.1 Participant instructions

1.1.1 Introductory instructions

Welcome to Planet Zonk! In this game, you will learn where alien plants grow.

Zonk is a donut-shaped planet. Every plant grows in a different place on Planet Zonk in summer vs winter. Your job as the planet naturalist is to learn where each plant grows in summer and winter. Take a look at some example plants below. On every play, you will be shown a plant and asked where it grows in a particular season. Once you have pressed Enter, you will see the true answer. [Practice trial].

Over time, you will learn the correct answers through trial and error. You might notice a pattern linking the seasons. After making your guess, you usually see the plant's true location as a colored circle. If the circle is red, you were not close and did not win any points. If the circle is green, you were close and won points. The closer you are to the true answer, the more points you win. Sometimes, you won't see the answer but you still get points if you were close. The total points you have won will be shown in the top right corner of the screen. At the end of the game, your bonus payment will be based on the number of points you win over the course of the game.

The number of plays remaining is shown at the top left. This number shows you the total questions left before you are done (note there are different sections within this). We will give you time for quick breaks, but please do not take longer than five minutes before moving on or the study will terminate automatically. Please do not use pen and paper—only your memory.

Note: You won't be able to learn about where the plants grow from what they look like! You will have to use trial and error to learn.

Are you ready? Press the forward arrow to start the task now. Please go through the instructions again if you are unsure about anything.

1.1.2 Re-test instructions

From now on, you will only see feedback on some trials, but you still get points every time you are close to the correct answer. Some of these plants you have not seen in a while. Try to remember where they all grow. Press space to continue.

1.1.3 Debrief instructions

Almost done! One last question. Some plants you saw at the beginning of the game. Other plants you only saw for the first time halfway through. Before you leave, we will ask you when you first saw each plant. Press space to continue.

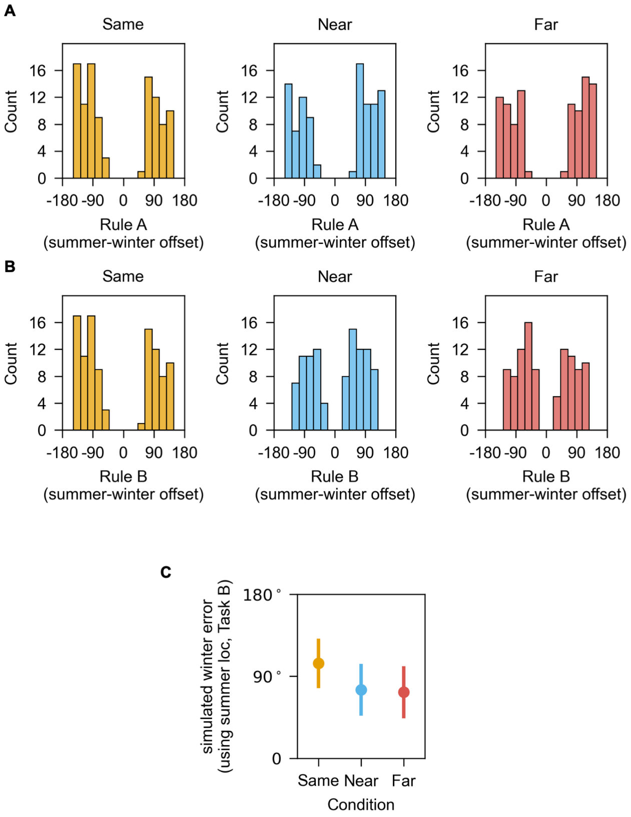

Figure S1: **Matched task rules across conditions.** (A) Histograms of Task A rules (i.e., the angular offset between summer and winter locations during Task A) for participants in the Same (yellow), Near (blue), and Far (red) conditions. Task A rules are matched across conditions. (B) Histograms of Task B rules across the same three conditions. Task B rules in the Near and Far conditions were identically distributed and offset by ±30° or 180° from their corresponding Task A rules. Task B rules in the Same condition remain within the original Task A range. (C) Mean expected error across conditions if participants had responded to winter trials by simply selecting the location where they had received summer feedback (during Task B). This simple strategy would produce larger errors in the Same condition (due to a wider average angular separation between summer and winter) and similar errors in Near and Far conditions. However, empirical results show the opposite pattern—suggesting that participants rely on learned task rules rather than a spatial proximity heuristic. Error bars indicate standard deviation.

2 Additional ANN analyses of transfer / interference

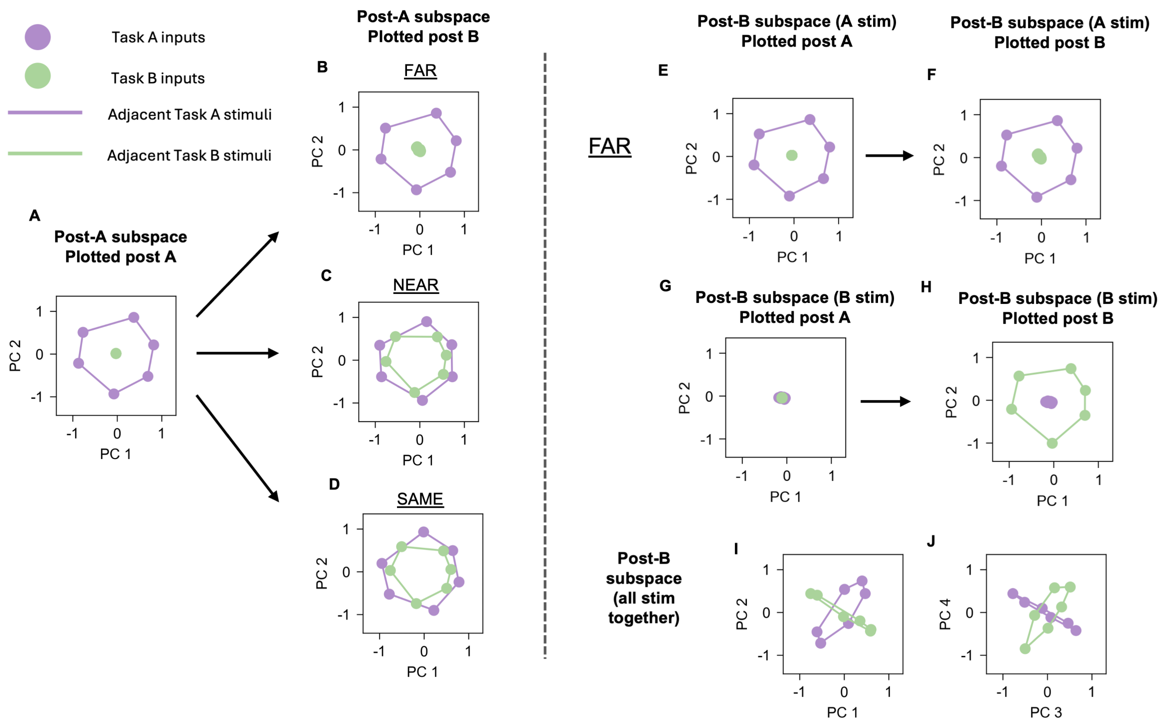

Figure S2: **Evolution of ANN hidden layer subspaces.** **(A)** Task A and Task B stimuli projected into the subspace defined by the first two principal components obtained after training on Task A. Task A stimuli are well-separated in this subspace, and in the Same and Near conditions, this subspace continues to be used after Task B training. **(B)** In the Far condition, Task B stimuli remain collapsed at the origin even after Task B training, indicating learning unfolds in a separate subspace. **(C)** In the Near condition, Task B stimuli reuse the Task A subspace. **(D)** Same as (C), but for the Same condition. **(E–H)** Subspace projections in the Far regime, confirming Task A and Task B occupy orthogonal subspaces. **(I–J)** PCA across all inputs post-Task B training in the Far regime shows the union of two orthogonal 2D subspaces spanning a 4D space.

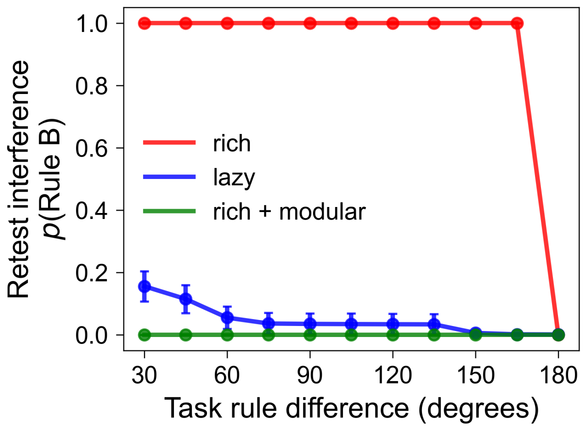

Figure S3: **Interference across a continuum of rule similarities.** Neural networks were trained on rule shift angles from 30° to 180° in 15° increments. For each of 10 participant-matched training schedules, networks were trained under three regimes: rich (red), lazy (blue), and rich + modular (green). The y-axis shows interference (probability of applying Rule B at Task A re-test) as a function of angular distance between task rules. Rich networks show a sharp nonlinearity in interference, transitioning to orthogonal representations only for dissimilar tasks. Lazy networks show consistently low interference. Modular networks eliminate interference across all angles due to enforced subspace separation.

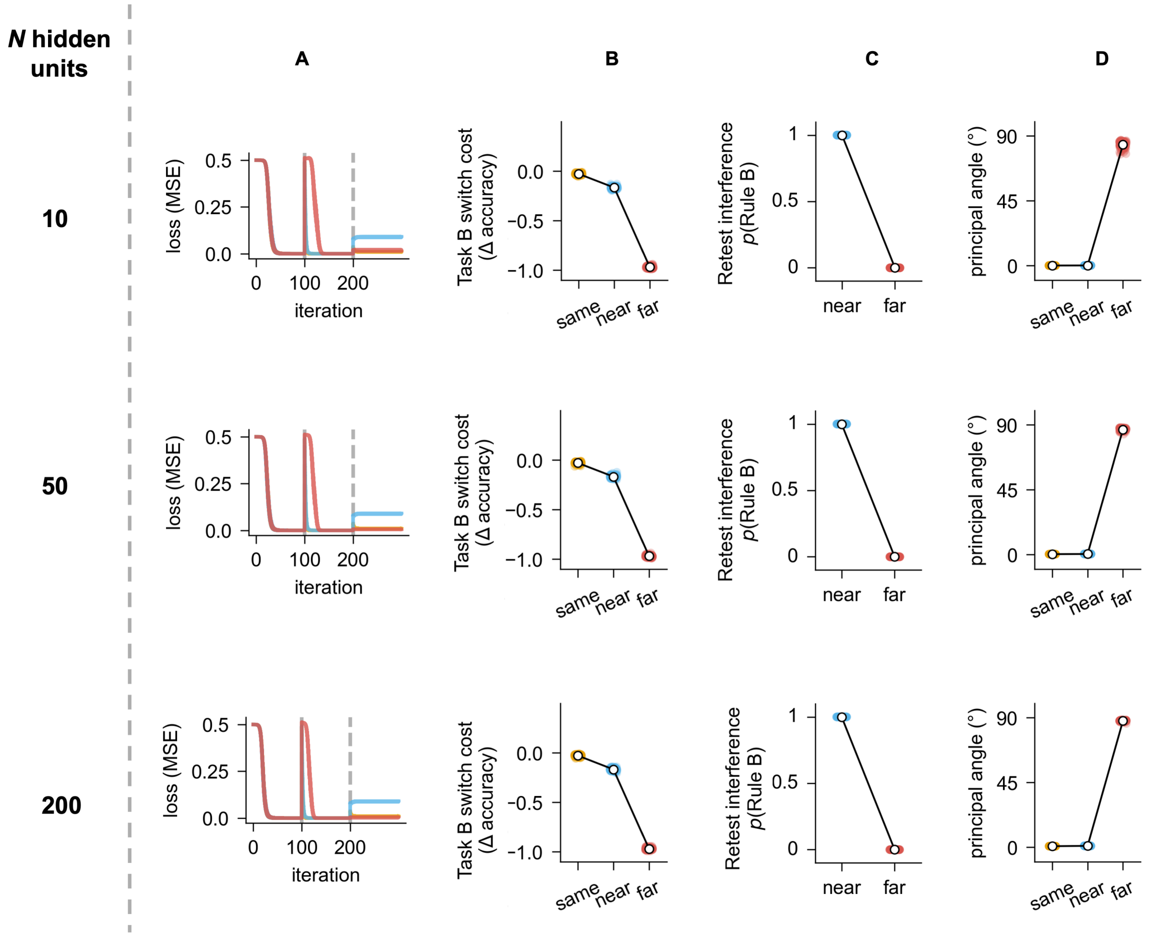

Figure S4: **Patterns of transfer and interference in ANNs do not depend on hidden unit size.** **(A)** Training loss curves for networks with 10, 50, and 200 hidden units across Same, Near, and Far conditions. **(B)** Transfer performance across unit sizes, measured as change in winter accuracy from final Task A to first Task B exposure. **(C)** Interference measured as probability of applying Rule B at Task A re-test. **(D)** Principal angles between Task A and Task B subspaces, with larger angles reflecting greater representational separation.

2.1 ANN implementation with non-linearities (ReLU)

To test whether our main findings generalized to non-linear networks, we trained networks with a ReLU layer in the Same, Near, and Far regimes. These networks followed a two-layer feed-forward architecture with a ReLU activation function applied to the hidden layer (**Fig. S5A**). The architecture followed an input → hidden → output structure, where both layers were fully connected and did not include bias terms. Formally:

$$h = \mathrm{ReLU}(W_1 \cdot x), \quad y = W_2 \cdot h$$

Here, x is the input vector, W_1 and W_2 are the input-to-hidden and hidden-to-output weight matrices, h is the hidden layer activation, and y is the output. Weights were initialized from a Gaussian distribution with mean 0 and variance $\sigma = 10^{-3}$, matching the rich regime initialization used in linear networks. The hidden layer had $n_h = 200$ units, as greater capacity was needed to ensure stable dynamics under the non-linear activation.

Our results generalised to this setting: Far condition networks showed reduced interference and worse transfer compared to Near condition networks (**Fig. S5B–D**). Representational geometry analysis before the ReLU non-linearity (**Fig. S5F–H**) revealed that Far networks formed

orthogonal subspaces, consistent with a 90° angle between Task A and Task B representations (**Fig. S5E**).

The main difference between ReLU and linear networks was the higher representational dimensionality in the former. Before applying the ReLU, more principal components were needed to explain 95% of the variance in hidden activity, indicating richer representational complexity (**Fig. S5I**).

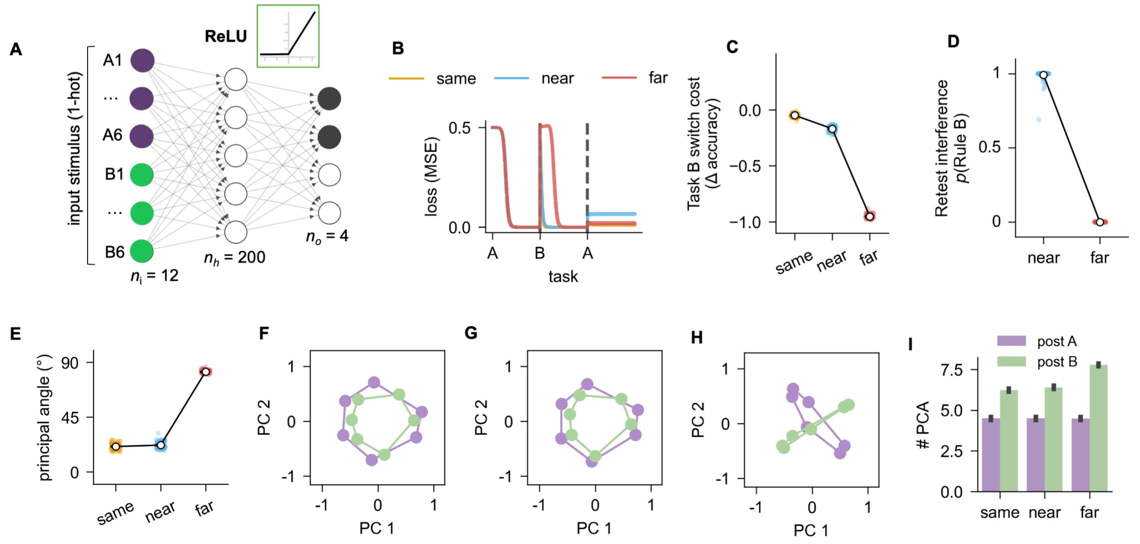

Figure S5: **Patterns of transfer and interference in a two-layer ReLU network.** **(A)** Network schematic with ReLU activation on the hidden layer. **(B)** Loss curves for Same, Near, and Far conditions. **(C)** Transfer to Task B. Far networks show reduced transfer. **(D)** Interference at Task A re-test. Far networks show reduced interference. **(E)** Principal angles between Task A and Task B subspaces post-training. Far networks show angles near 90°, indicating orthogonal subspaces. **(F–H)** PCA projections for Same, Near, and Far conditions (at the hidden layer, before reLU activation). Task A inputs in purple; Task B in green. **(I)** Dimensionality of the hidden layer representation (number of PCs explaining 95% of variance, pre-ReLU). ReLU networks exhibit higher complexity overall, with the greatest increase in Far condition.

3 Behavioural Effects of Task Similarity

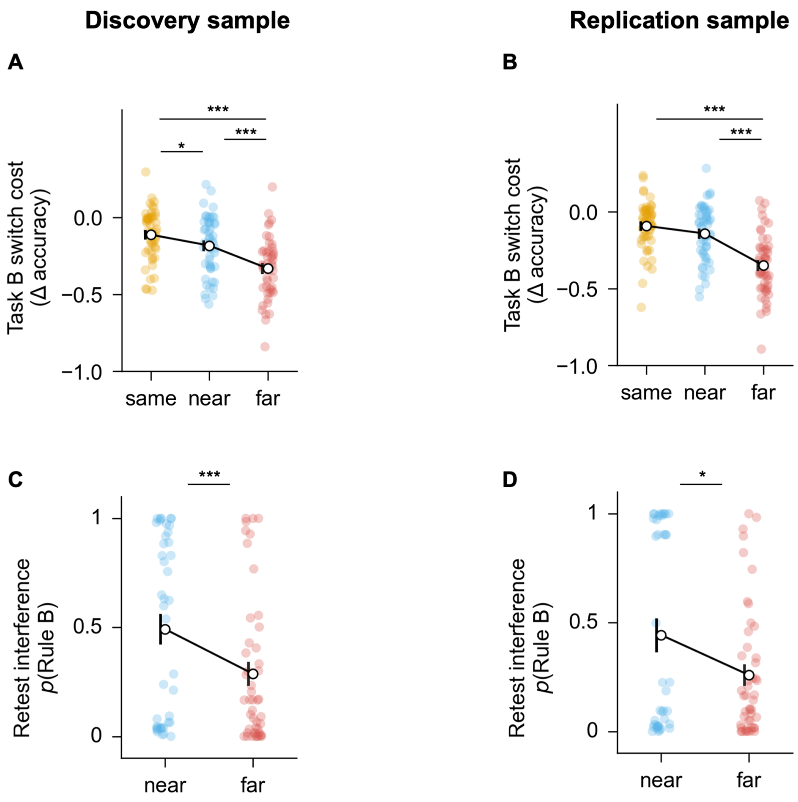

Figure S6: **Main results separated by Discovery and Replication sample.** (Discovery sample: Near, $N = 50$, Far, $N = 50$, Same, $N = 52$; Replication sample: Near, $N = 51$, Far, $N = 51$, Same, $N = 52$) **(A,B)** Transfer is the change in accuracy for the winter response after new stimuli are introduced at Task B (difference between winter accuracy in the final block of training on Task A, and the first block of training on Task B). Circles indicate mean, error bars show s.e.m. across the participant sample, colours correspond to condition. One-way Anova for effect of condition on transfer; Discovery sample: $F(2, 148) = 18.68$, $p < 0.001$; Replication sample: $F(2, 151) = 29.34$, $p < 0.001$. One-sided t-tests for the preregistered hypotheses showed that in both samples, Δ accuracy in the Far condition was significantly lower than the Near and Same condition; all $p < 0.001$; however, Δ accuracy in Near was significantly lower than in Same in the Discovery sample: $t(99) = 1.99$, $p = 0.025$, but not in the Replication sample: $t(101) = 1.47$, $p = 0.071$. **(C,D)** Interference is the probability of applying Rule B when retested on Task A. Circles indicate mean, error bars show s.e.m. across the participant sample, colours correspond to condition. Near>Far, one-sided t-test; Discovery sample: $t(86) = 2.56$, $p = 0.006$; Replication sample: $t(84) = 2.27$, $p = 0.012$.

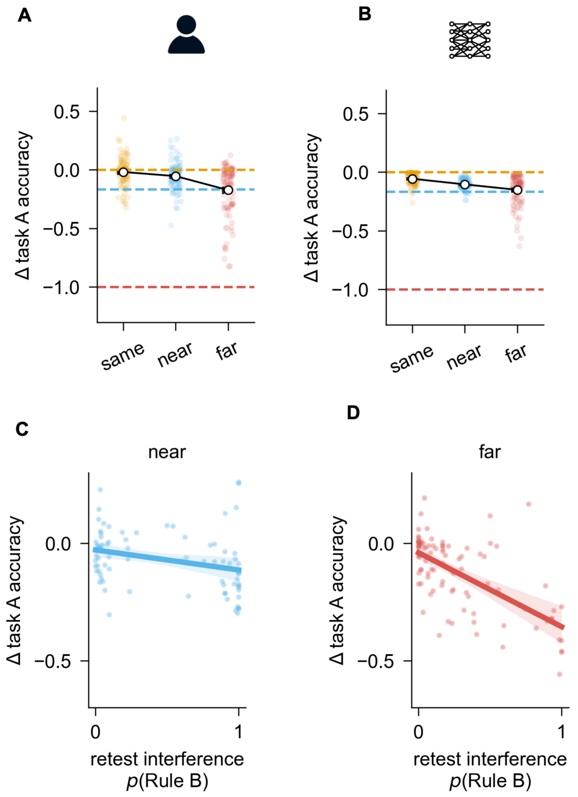

Figure S7: **Retest accuracy.** **(A)** Here we plot the average difference between training and re-test accuracy, namely winter accuracy in the final block of training on A minus winter accuracy during re-test of Task A. Accuracy change is plotted by condition. Dotted lines show the accuracy that would be expected if people updated to using Rule B in each condition – i.e. total catastrophic forgetting. This pattern of re-test error is captured by ANNs trained with unreliable stimulus identification, which we have reason to believe is the case from the identification test at the end (see B). **(B)** Same as (A) for ANNs trained with unreliable stimulus identification. Specifically, we recreate the possibility that learners mistake a Task A stimulus for a Task B stimulus during re-test, based on the empirical distribution of task swap errors that participants make when tested at the end of the study. For each network, we take the corresponding participant's probability of incorrectly classifying a stimulus as belonging to the opposite task during the debrief. During re-test on Task A, we use this as the 'swap probability' – the chance that the Task A one-hot vector will be swapped with the corresponding Task B input. **(C)** In the Near group of participants, interference (probability of applying Rule B during re-test) is inversely correlated with retest accuracy (Spearman's $r = -0.44, p < 0.001$). However, since the rules are by definition closer in Near, even during maximum interference the effect on accuracy is minimal compared to in the Far condition. **(D)** In the Far group of participants, interference (probability of applying Rule B during re-test) is also inversely correlated with retest accuracy (Spearman's $r = -0.78, p < 0.001$). However, even though significantly fewer individuals suffer from interference in the far condition compared to the near condition, these individuals have a disproportionate influence on average group accuracy because the effect on accuracy from swapping rules in the far condition is more catastrophic.

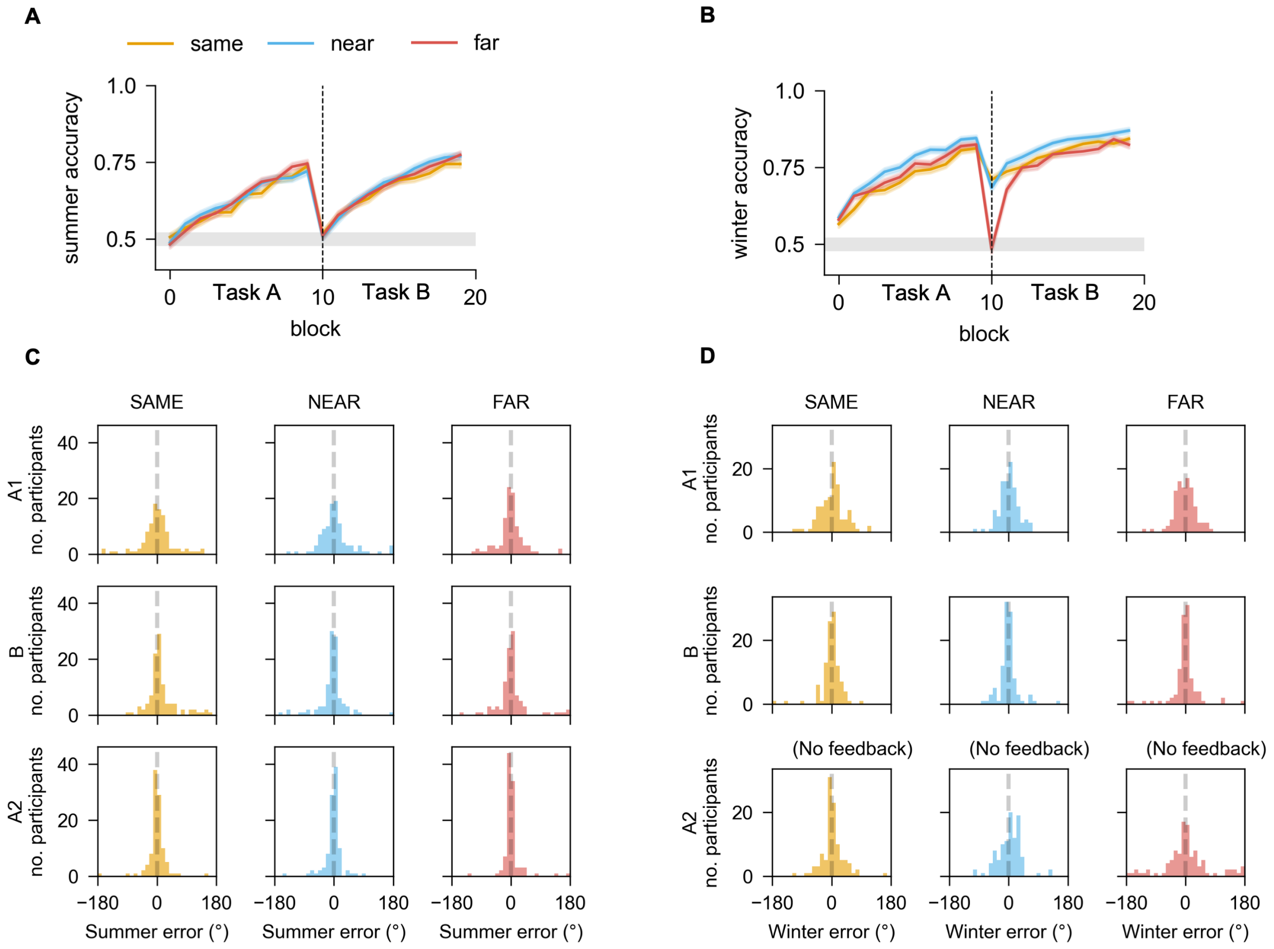

Figure S8: **Raw accuracy split by response type. (A)** Average summer accuracy during training of Task A and Task B. Note that an accuracy of 1 is equivalent to a perfect response (no error), while an accuracy of 0 is equivalent to 180° (maximum error). Dotted line depicts the transition from Task A to Task B, where new stimuli and the condition-dependent change in rule are introduced. Grey shaded bar shows chance performance. Coloured lines show accuracy split by condition, with shaded intervals showing s.e.m. **(B)** Same as (A) for winter response accuracy, where participants can leverage their knowledge of the structural rule. **(C)** Summer mean circular error across task sections and conditions. Histograms showing the distribution of summer mean circular error averaged across participants. Rows correspond to task sections–training on A (A1), training on B, and re-test on A (A2), plotted top to bottom. Columns correspond to conditions: Same (yellow), Near (blue), and Far (red). As can be seen, summer locations are effectively learned across all groups, with error decreasing across successive training phases. Error continues to decrease in A2, when participants continue receiving feedback on summer responses even though feedback for winter responses has been removed in this section (to test interference). **(D)** Same as (C) for winter responses.

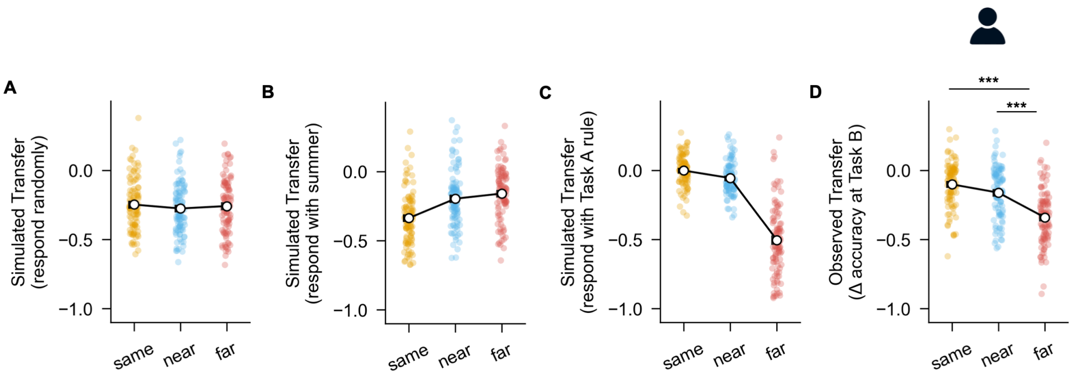

Figure S9: **Simulated transfer under alternative strategies.** Transfer (i.e., switch cost) is defined as the drop in accuracy from the final set of winter responses for Task A stimuli to the first set of winter responses for the Task B stimuli. **(A–C)** Simulated switch costs for three alternative strategies. **(A)** Random responding – winter responses in Task B are drawn from a uniform distribution. **(B)** Summer-repeat strategy – winter responses in Task B replicate the preceding summer feedback. This strategy predicts lower accuracy in summer due to control constraints that matched the Task A rules across all conditions, while the rules for Near and Far were also matched in Task B (see Figure S1). **(C)** Rule-based strategy – participants continue applying the Task A rule to the feedback they receive on summer for the new Task B stimuli. To capture participant-specific variability, we fitted a von Mises distribution to each participant's winter responses in the second half of Task A (blocks 5–10), using the known Task A rule as the fixed mean and estimating individual κ. This precision was used to simulate variable rule-based responses in Task B. **(D)** Human behavioural data showing switch costs across task conditions. Only the rule-based strategy (C) reproduces the condition-specific switch cost pattern observed in the human data–namely, greater transfer in the Same and Near conditions than in the Far condition. Error bars denote s.e.m. across participants.

4 Model Fitting

4.1 Interference Model Fitting

4.1.1 EM Algorithm for Mixture of von Mises Distributions

To quantify interference, we fit a mixture of von Mises distributions [1] capturing the use of Rule A and Rule B to participants' responses. First, we calculated the rule used for each winter trial by subtracting the participant's winter response from the feedback for the previous summer response. Then, we fit the rule responses to a mixture of von Mises distributions (i.e., the circular analogue of the normal distribution) with pre-determined means of Rule A (θ_A) and Rule B (θ_B). This model had two free parameters: a mixing weight (π) capturing the relative contribution of θ_A and θ_B, and a single concentration parameter (κ) representing dispersion of the distributions around their respective means.

To fit the mixture model, we used an expectation-maximization (EM) algorithm, iterating over a range of initial parameter values to identify the best-fitting model based on log-likelihood. We tested π values ranging from $p(\theta_B) = 0.1$ to $p(\theta_B) = 0.9$, in increments of 0.1. We tested κ values 1, 2.5, 5, and 10. This ensured a comprehensive exploration of the parameter space, accounting for different initialisations. To fit the distributions using EM, we wrote custom Python code adapted from `https://framagit.org/fraschelle/mixture-of-von-mises-distributions` [2], based on the method presented in [1].

For each initial parameter set, the algorithm iteratively optimized the parameters until convergence (where convergence was met when the parameter update fell below a threshold of 1×10^{-3}). On each iteration, the algorithm estimated the probability of data belonging to each distribution, to calculate the updated value of π (i.e., the probability density function of each data-point given the current π and κ, normalized across the two distributions). The concentration parameter (κ) was updated subsequently by minimizing the negative log-likelihood using the L-BFGS-B optimization algorithm. This process was iterated until convergence.

After fitting the model for all combinations of initial parameters, we selected the best-fitting model based on the log-likelihood of the observed data. The initialization set that yielded the highest log-likelihood was chosen as the optimal solution. This approach allowed us to avoid local minima and ensure robust parameter estimation.

4.1.2 Interference Sanity Checks

We defined interference at re-test of Task A as π, which is the probability of responding with Rule B ($p(\theta_B)$). Our main analysis tested whether interference was greater in the Near condition than the Far condition, using a one-sided t-test.

To exclude the possibility that the higher interference in the Near condition was driven by differences in initial learning of Task A, we also quantified the *change* in interference weight between training and re-test of Task A (fitting the model separately to Task A training and Task A re-test). To calculate this, we fit the mixture of von Mises models separately to training in A (excluding the first block to allow time for learning), and to the re-test period. We then calculated the change in use of Rule B as $\Delta p(\theta_B) = \pi_{retest} - \pi_{training}$, and performed a one-sided t-test to test that $\Delta p(\theta_B)$ was greater in the Near condition than the Far condition. This confirmed that the use of Rule B increased more for participants in the Near condition than the Far condition as a result of learning Task B (Δp(Rule B) in Near > Far, one-sided t-test; Discovery sample: $t(86) = 1.94$, $d = 0.42$, $p = 0.028$; Replication sample: $t(84) = 2.20$, $d = 0.48$, $p = 0.015$).

4.1.3 Parameter Recovery and Permutation Test Validation

To assess the recoverability of the parameters, we simulated 10 datasets per participant schedule, on each iteration shuffling the empirical values found for π and κ. All parameters showed good recoverability: The Pearson correlation between simulated and recovered values of π and κ were 0.87 and 0.98 respectively in the Near condition, and 0.96 and 0.97 respectively in the Far condition.

Finally, we performed a permutation test analysis to confirm that the difference between Near and Far group parameters could not be explained by a difference in the recoverability of the π parameter in these groups (mixing weight parameter capturing interference from Rule B, or $p(\theta_B)$). Across 1000 iterations, we randomly shuffled the parameters across all participants. On each iteration, we simulated data with the shuffled parameters, and recovered the parameters, caching the difference between the average π recovered in the Near and Far conditions. This resulted in a distribution of differences in π between the Near and Far groups due to chance. The 95th percentile of this distribution was a difference of 0.11 (while the empirical value = 0.12). The probability of seeing the same size interference effect due to differences in recoverability between conditions is therefore $< 5\%$.

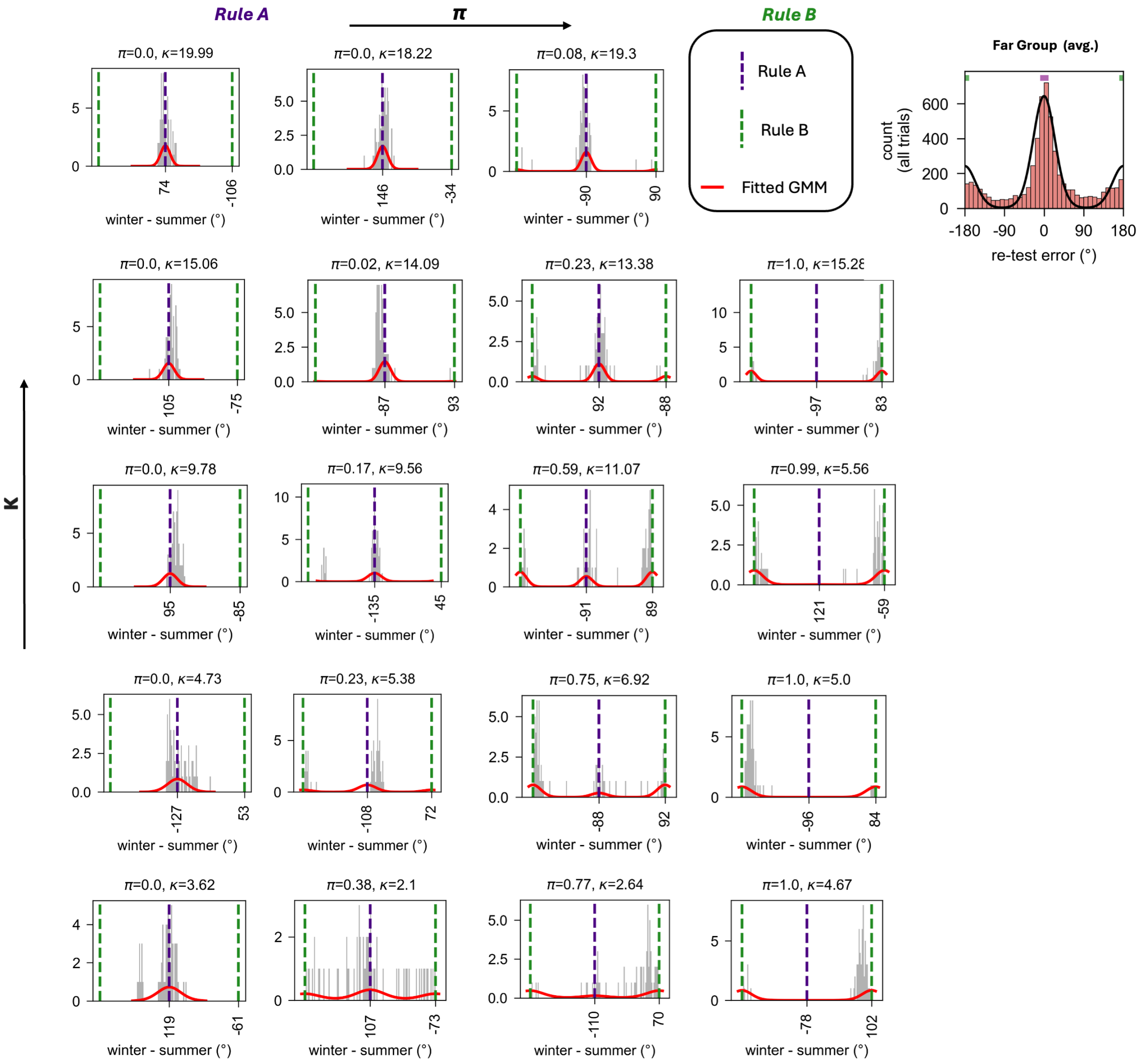

Figure S10: **Participant response histograms and model fits (Far)**. Individual participant response histograms and fitted model posteriors in the Far group. Participants were selected to cover a range of π and κ values to visualise goodness of fit. Each subplot shows a histogram of winter responses for a selected individual participant from the Far condition, plotted as the angular distance from the summer feedback (reflecting how the participant applied the task rule). The purple dotted line marks the expected response under Rule A; the green dotted line marks the expected response under Rule B. The red line shows the posterior of the fitted mixture of von Mises model. Each subplot is annotated with the participant's fitted mixture weight (π) and concentration parameter (κ). Plots are sorted by increasing κ (bottom to top) and increasing π (left to right). This visualization illustrates the range of individual strategies observed within the Far condition, as well as the quality of fit achieved by the mixture of von Mises model across selected participants.

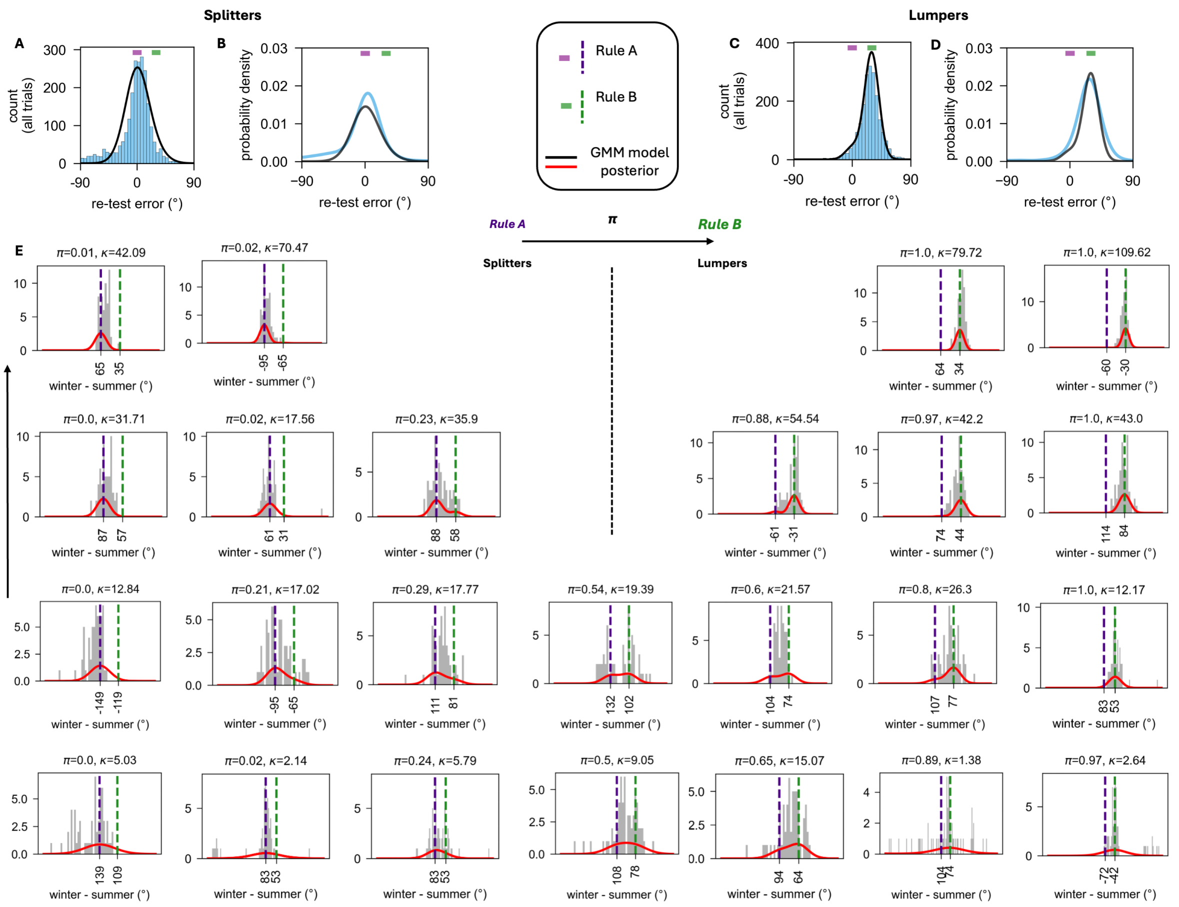

Figure S11: **Participant response histograms and model fits (Near).** Individual response distributions and model fits in the Near group. (A) Splitters: aggregated histogram of re-test errors across all participants (blue), with fitted model posterior probability (black line; scaled by participant numbers). (B) Splitters: kernel density estimate (KDE) of the empirical distribution (blue) overlaid on the model posterior probability density (black). (C) Lumpers: aggregated histogram of re-test errors (blue), with fitted model posterior probability (black). (D) Lumpers: KDE of the empirical distribution (blue) overlaid on the model posterior probability density (black). (E) Individual participants: histograms of winter responses, plotted as angular distance from summer feedback (reflecting application of the task rule). The purple dotted line marks the expected response under Rule A; the green dotted line marks the expected response under Rule B; the red line shows the fitted mixture of von Mises posterior. Each panel is annotated with fitted mixture weight (π) and concentration parameter (κ). Participants are ordered by increasing κ (bottom to top) and π (left to right). Participants to the left of the central dotted line (low π) are classified as splitters (predominantly Rule A responses), while those to the right (high π) are lumpers (predominantly Rule B responses). At the top of each group, the average response distribution across participants is shown with the average fitted model posterior overlaid. This visualization illustrates both the diversity of individual strategies and the quality of the model fits across participants.

4.2 Characterizing Interference Errors: Swaps vs. Biases

To better understand the nature of interference during Task A re-test, we asked: what kinds of errors do participants make when interference occurs? Specifically, are these errors best explained by a single distribution that may arise from a gradual shift away from the original rule (i.e., a Task B bias), or by binary switch errors to the Task B rule (i.e., swaps)? This distinc-

tion is important because it may reflect different underlying computational processes–such as incremental updating of a single representation versus the formation of distinct latent causes.

Our predictions about the nature of interference errors are motivated by the geometry of representations observed in artificial neural networks (ANNs). In the *Same* and *Near* conditions, task inputs are encoded using overlapping representational subspaces (e.g., shared principal components), such that both tasks are embedded within a common latent space. In contrast, the *Far* condition results in orthogonal representations across tasks. If humans rely on similar representational structures, their interference patterns should mirror this geometry. Specifically, in the *Near* condition, interference errors (among 'lumpers') should arise from updating a single distribution (e.g., gradual interpolation). In the *Far* condition, we would expect errors to reflect mis-classification of a stimulus' task identity (i.e., swap-like behaviour).

To test these predictions, we implemented a model comparison framework to distinguish between swap and bias dynamics. Each participant's distribution of responses at re-test (on Task A stimuli) was fit using five candidate models:

- **Uniform**: A control model capturing responses consistent with pure noise (no free parameters).
- **Rule A**: Responses drawn from a single von Mises distribution centered on the original Task A rule (1 fitted κ).
- **Rule B**: Responses drawn from a single distribution centered on the new Task B rule (1 fitted κ).
- **Interpolated**: Responses drawn from a weighted average of Rule A and Rule B means, capturing graded updating of a shared distribution (1 interpolation weight, 1 κ).
- **Mixture**: A mixture of von Mises distributions centered on Rule A and Rule B, capturing trial-level swaps between rules (1 mixture weight π, 1 shared κ).

We compared models using Bayesian model selection for group studies [3, 4], implemented via the `bms` package [5]. This analysis yielded results consistent with our ANN predictions. In the *Far* condition, the mixture model had the highest protected exceedance probability, consistent with swap-like errors and inference of separate latent causes. A subset of participants in this condition was best fit by the Rule A model, consistent with minimal interference.

In the *Near (lumpers)* group, the best-fitting model was Rule B, suggesting that these participants updated a single internal distribution to reflect the new Task B rule. In the *Near (splitters)* group, the best-fitting model was Rule A, indicating resistance to interference. Notably, in both Near subgroups, a proportion of participants were better explained by the interpolation model, consistent with graded updating within a shared latent context. Crucially, the mixture model was rarely preferred in either Near group, indicating minimal evidence for swap-like interference. The model comparison results are shown in Supplementary Figure S12.

4.3 Model Variant: Fitting Interference Offset

To further investigate the nature of interference in the *Near (lumpers)* group, we tested whether the interference component of the response distribution could be better captured by allowing its mean direction to be freely estimated, rather than fixed to the Task B rule.

We fit a shifted von Mises model, where each response θ_i was modelled as a deviation from Rule A, plus a free 'interference' offset:

$$\theta_i \sim \text{von Mises}(\mu = \mu_A + \Delta, \kappa) \qquad (1)$$

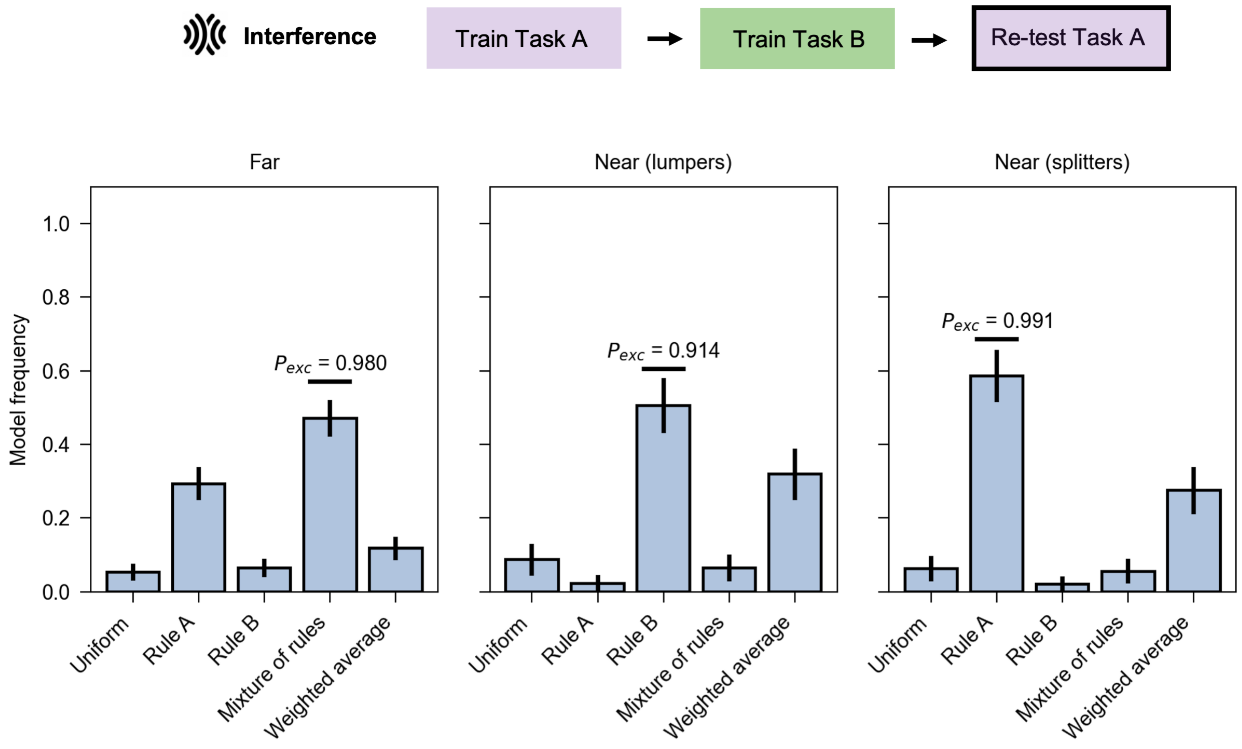

Figure S12: **Model comparison results distinguishing between swap-like and bias-like interference errors.** Each participant's responses at re-test on Task A stimuli were fit using five candidate models: a uniform model (control), Rule A model (original rule), Rule B model (fully updated to new rule), an interpolated rule model (shifted mean), and a mixture model (trial-level swaps between rules). Bars indicate the expected model frequency within each group (Far, Near (lumpers), Near (splitters)) as estimated by Bayesian model selection for group studies [3, 4], implemented via the `bms` package [5]. Error bars reflect the standard deviation of the Dirichlet distribution over model frequencies. Horizontal lines and annotations indicate the winning model in each group, with associated protected exceedance probability (P_{exc}).

Here, μ_A is the known mean direction of Rule A, Δ is a free offset parameter capturing the shift in mean response direction, and κ is a concentration parameter. Parameters were estimated via maximum likelihood.

The resulting fitted Δ values are plotted in Supplementary Figure S13, colour-coded by the true Rule B offset from Rule A. We observed a strong correspondence between the fitted offsets and the ground truth Rule B directions, consistent with interference in this group primarily reflecting full updating to Rule B. This supports the interpretation that *lumpers* in the *Near* condition primarily update a single rule representation during Task B, rather than swapping between discrete rules.

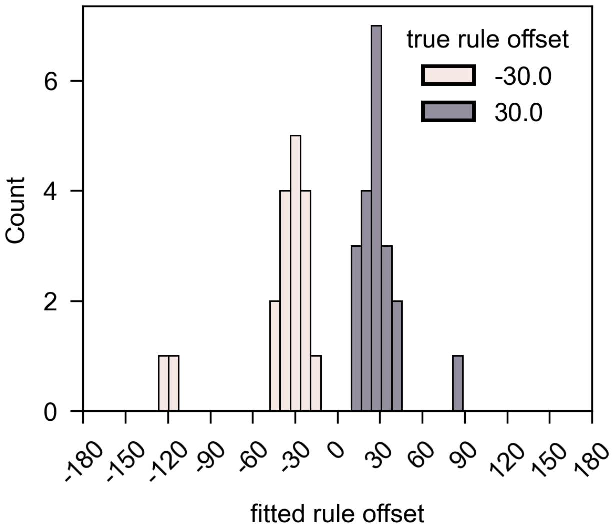

Figure S13: **Fitted interference weight for Lumpers.** Fitted offset of responses from the original Task A rule, for participants in the Near (lumpers) group. Each bar represents a participant's fitted Δ value from a von Mises model allowing a free mean shift from Rule A. Bars are colour-coded by the true angular offset between Rule A and Rule B (either $-30°$ or $+30°$).

5 Individual Differences

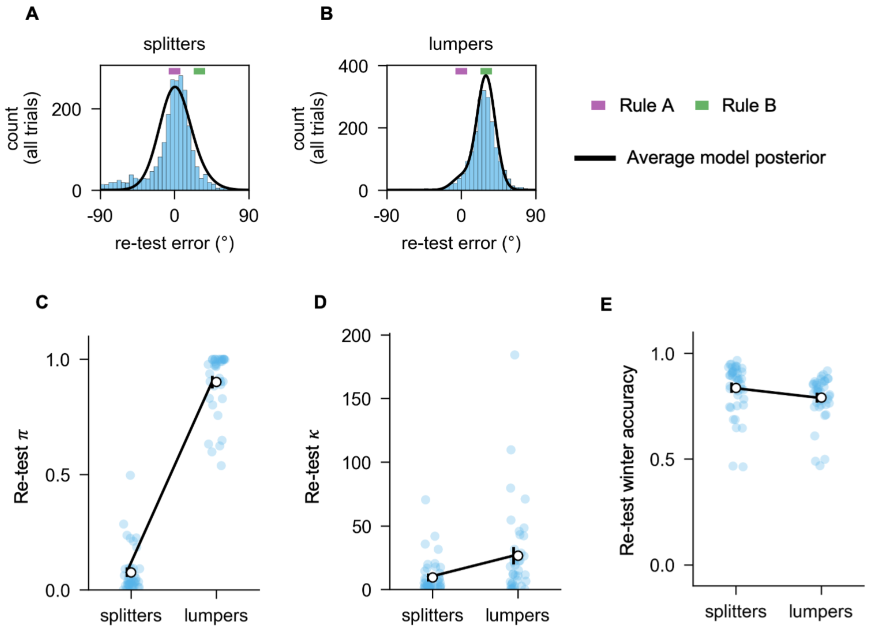

Figure S14: **Comparing response distributions in lumpers and splitters. (A–B)** Distribution of all retest response errors pooled across participants for lumpers (left) and splitters (right), with the average posterior of the fitted mixture of von Mises model overlaid (black line). Purple and green notches mark the expected error if applying the Task A or Task B rule, respectively. Lumpers cluster around the Task B rule, while splitters are centered on the Task A rule. The distribution for splitters is broader, indicating lower precision. **(C)** Mixture model weight (π) for splitters versus lumpers. Circles show group means, dots represent individual participants, and error bars indicate s.e.m. **(D)** Same as (C), but for fitted κ (kappa) of the von Mises distribution. Lumpers show higher κ, reflecting greater response precision ($t(78) = 2.83$, $p = .0059$; Mann–Whitney $U = 1098.0$, $p = .0039$). **(E)** Average retest winter accuracy for each group. Despite higher κ values, lumpers show lower accuracy–consistent with the interpretation that they apply the Task B rule consistently but inappropriately during re-test. Splitters, by contrast, show more variable responses (lower κ) but higher accuracy, suggesting a strategy that better preserves the original Task A mapping during Task B learning ($t(78) = -1.80$, $p = .076$; Mann–Whitney $U = 512.0$, $p = .0059$).

5.1 Accuracy Across Training in Lumpers, Splitters, Rich, and Lazy Networks

In the main text, we report that splitters outperform lumpers in summer accuracy during Task A ($t(78) = 3.40$, $p = 0.001$). Here, we show that this effect generalizes across the entire experiment (Fig. S15A): splitters also show higher summer accuracy during Task B ($t(78) = 4.11$, $p < 0.001$), during A re-test ($t(78) = 3.95$, $p < 0.001$), and when averaging across all phases ($t(78) = 4.44$, $p < 0.001$).

Differences in summer accuracy during Task A are also observed in neural networks (Fig. S15B): lazy networks exhibit significantly higher summer accuracy than rich networks during Task A ($t(200) = 3.60$, $p < 0.001$). This arises because lazy networks are initialized with larger weights, enabling faster convergence as they form high-dimensional random projections [6**?**]. Crucially,

when we match the number of training trials between humans and ANNs (first 120 trials of Task A), the behavioural profiles are aligned: lumpers resemble rich networks and splitters resemble lazy networks.

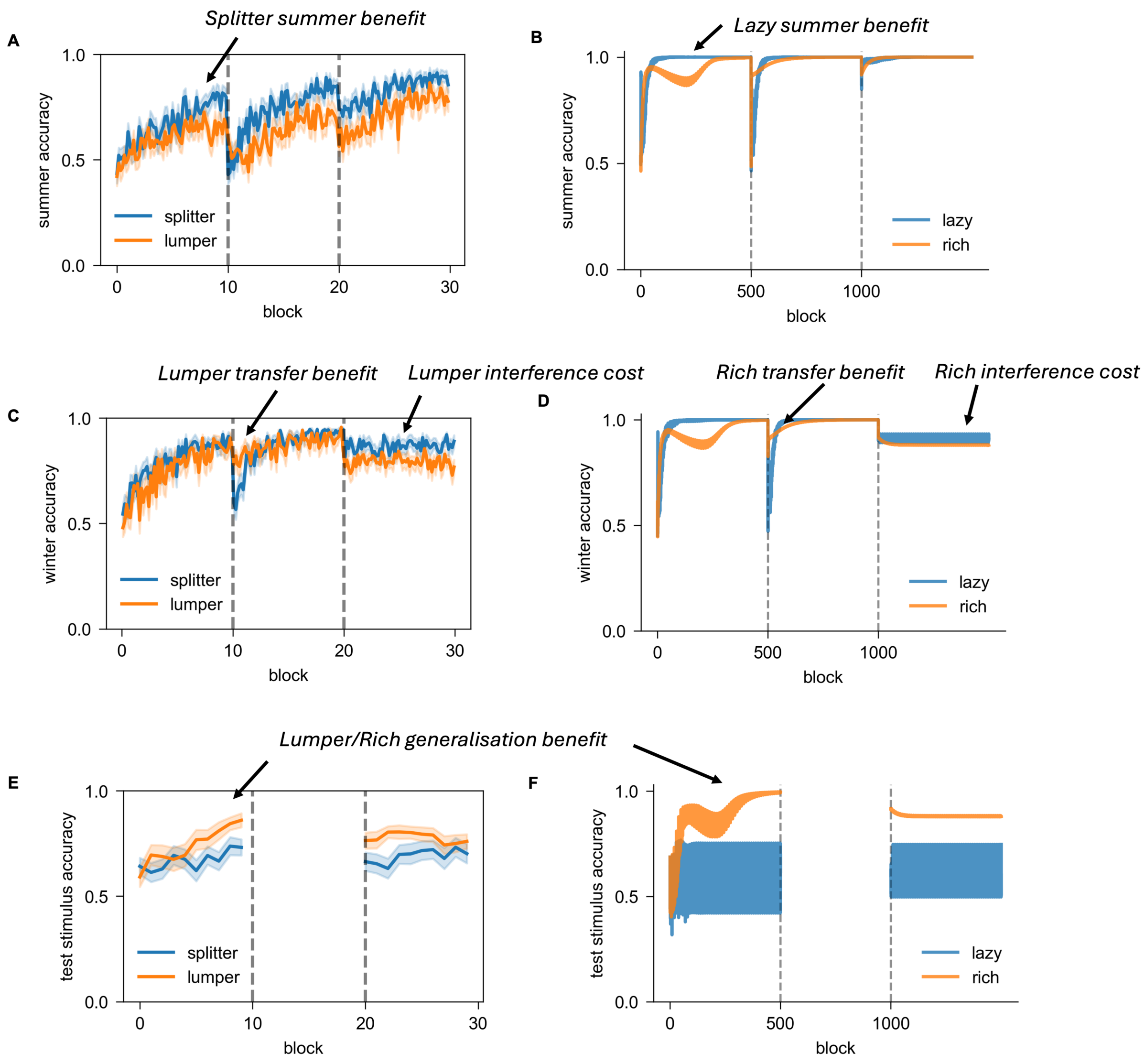

Figure S15: **Training accuracy for splitters vs. lumpers and rich vs. lazy networks.** **(A)** Summer accuracy for human participants in the Near condition, averaged across blocks and separated by group: lumpers (orange) and splitters (blue). Shaded areas represent ± 1 SEM. Vertical dotted lines indicate transitions between Task A, Task B, and A re-test. **(B)** Same as (A), but for ANNs trained in the lazy (blue) or rich (orange) regime. Here we show ANN performance on the first 50 repetitions of their participant-matched schedule per task phase. While networks were trained for 100 repetitions, only the initial 50 are shown to highlight the learning trajectories prior to convergence, as performance in the final 50 repetitions had already plateaued. **(C)** Winter accuracy for the same human participants shown in (A). **(D)** Winter accuracy for the corresponding ANNs shown in (B). **(E)** Winter accuracy for the test stimulus, where participants receive summer feedback only and must infer the correct winter location. **(F)** Winter accuracy for the test stimulus in corresponding ANNs. No gradient updates are performed for the test stimulus winter trials.

5.2 Excluding Random Responders

To ensure that our findings concerning differences in behaviour between Splitters and Lumpers were not confounded by participants responding randomly, we implemented a model-comparison procedure using leave-one-out cross-validation (LOO-CV). Specifically, we compared the goodness-of-fit of participants' response distributions under task-relevant von Mises models versus a uniform distribution.

For each participant in the *Near* condition, we estimated the predictive log-likelihood of their winter responses under three candidate models: (i) a von Mises distribution centered on rule A, (ii) a von Mises distribution centered on rule B, and (iii) a uniform distribution over the circular space. The von Mises models had concentration parameter κ fit separately for each participant. To evaluate predictive performance, we used LOO-CV: each data point was held out in turn, κ was re-fit on the remaining $N-1$ data points, and the log-likelihood of the held-out response was computed. Summing over all folds yielded a LOO-CV log-likelihood for each model.

The uniform model required no fitting: its log-likelihood is constant, given by

$$\mathrm{LL}_{\mathrm{Uniform}} = N \cdot \log\left(\frac{1}{2\pi}\right).$$

Participants were flagged as *random responders* if the uniform model provided a higher LOO-CV log-likelihood than both von Mises models during re-test of task A, i.e.

$$\mathrm{LL}^{\mathrm{LOO}}_{\mathrm{Uniform}} > \mathrm{LL}^{\mathrm{LOO}}_{\mathrm{RuleA}} \quad \text{and} \quad \mathrm{LL}^{\mathrm{LOO}}_{\mathrm{Uniform}} > \mathrm{LL}^{\mathrm{LOO}}_{\mathrm{RuleB}}$$

We then repeated our main individual difference analyses after excluding these participants. Importantly, all main findings remained unchanged, confirming that our results are not driven by the presence of random responders.

In the Near condition, we initially identified 42 Splitters and 38 Lumpers. After excluding participants classified as random responders using the LOO-CV uniform model criterion, 40 Splitters and 35 Lumpers remained. All key results remained significant and in the same direction. Specifically, lumpers continued to demonstrate superior transfer compared to splitters (two-sided t-test: $t(73) = 3.81$, $p < 0.001$, $d = 0.89$, 95% CI[0.07, 0.24]), as well as greater generalisation accuracy (two-sided t-test: $t(73) = 3.02$, $p = 0.003$, $d = 0.71$, 95% CI[0.05, 0.23]). Conversely, splitters continued to show higher summer accuracy than lumpers (two-sided t-test: $t(73) = 2.99$, $p = 0.004$, $d = 0.69$, 95% CI[0.022, 0.108]).

At re-test, splitters continued to show lower response precision than lumpers (concentration parameter κ; two-sided t-test: $t(73) = 3.00$, $p = 0.004$, $d = 0.68$, 95% CI[5.6, 31.9]), alongside higher accuracy (two-sided t-test: $t(73) = 2.16$, $p = 0.034$, $d = 0.51$, 95% CI[0.004, 0.074]). Finally, lumpers remained significantly worse at end-of-study categorisation test for Task A and Task B stimuli, indicating poorer temporal separation of the tasks (two-sided t-test: $t(73) = 3.56$, $p = 0.001$, $d = 0.81$, 95% CI[6.09, 22.99]).

Together, these results confirm that all main findings are robust to the exclusion of participants whose responses were better fit by a uniform distribution (responding randomly).

6 Mitigating Interference in ANNs

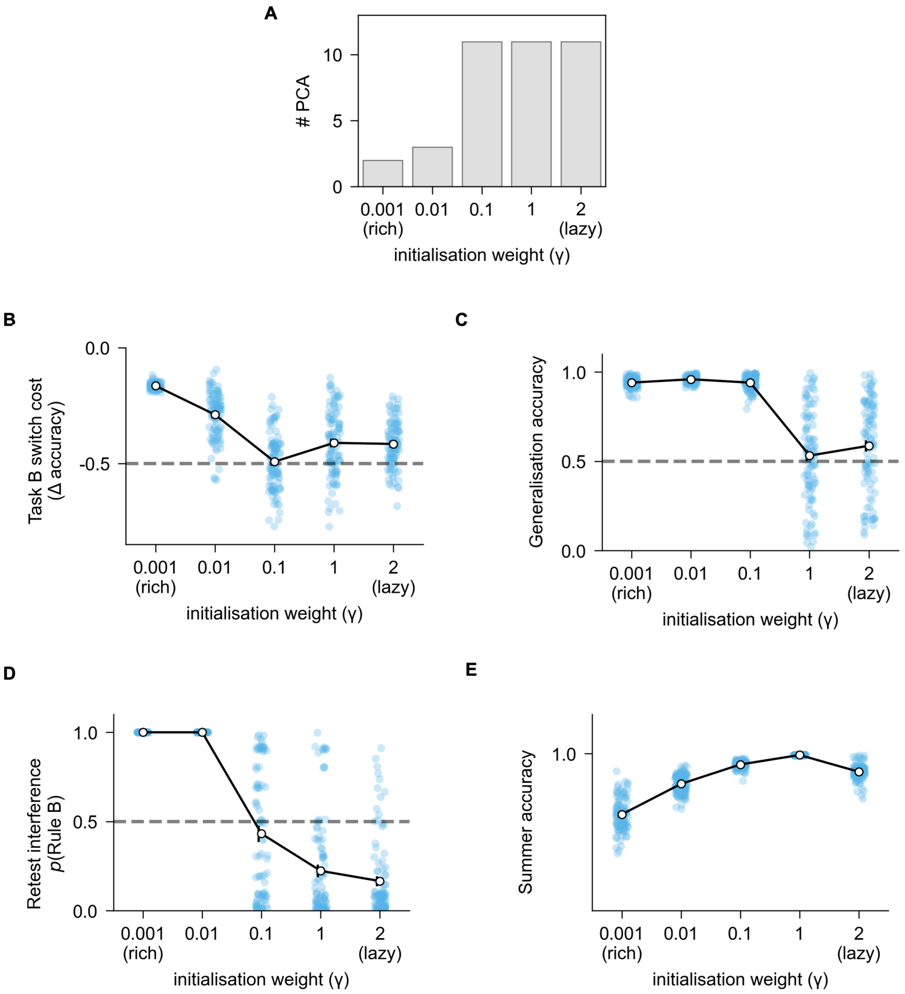

Figure S16: **Continuum of rich to lazy learning.** Behaviour in ANNs trained on a continuum from the rich regime (low weight initialisation) to the lazy regime (high weight initialisation). In each plot, the x-axis depicts the variance of the embedding weights at initialisation (γ). Smaller initial weights result in the network converging on low-dimensional solutions that lead to structured representations of the relevant information (*rich*). In contrast, higher initial weights result in the network converging on high-dimensional solutions where the inputs are expanded through random projections to the network hidden layer [6–15]. All network weights were initialised as random samples from Gaussian distributions with a mean of zero, and a variance of γ. **(A)** The dimensionality of the solution is quantified as the number of principal components needed to account for 99% hidden layer variance, after training is completed. As the initialisation weights increase, the dimensionality of the representation also increases. **(B)** Transfer to Task B decreases with larger weight initialisations. Transfer is the change in winter accuracy between the final block of Task A and the first exposure to Task B stimuli. **(C)** Generalisation is the winter accuracy for the test stimulus in Task A, for which no gradient updates are performed. **(D)** Interference from Task B decreases for larger weight initialisations. **(E)** Average accuracy for the summer response is higher in lazy regimes. In ANNs, this can be explained by differences in training speed, where lazy regime networks converge faster than rich regime networks.

6.1 Mitigating Interference: EWC, Replay, and Modular Architectures

To explore alternative mechanisms for mitigating interference in Splitters, we implemented three widely studied approaches: Elastic Weight Consolidation (EWC), replay, and modular architectures.

Our motivation was twofold: (1) to compare the behavioural signatures that emerge from different interference-mitigation strategies; and (2) to evaluate how these match the behavioural profiles of our "splitters"—participants who avoid interference in the Near condition.

6.2 EWC

Elastic Weight Consolidation (EWC) mitigates catastrophic forgetting by selectively constraining changes to parameters that are important for previously learned tasks, based on their estimated contribution to task performance. To implement EWC, we first trained the network on Task A and saved the resulting parameter values θ_A^*. We then estimated the diagonal of the Fisher Information Matrix to quantify the importance of each parameter for Task A. Specifically, we accumulated squared gradients of the loss across multiple stochastic forward passes through Task A data, introducing small amounts of Gaussian noise to the labels to stabilise the gradient estimates. The resulting Fisher estimates were averaged over passes and scaled by a constant factor.

During Task B learning, we added an EWC penalty to the standard loss function to discourage deviations from important Task A parameters. For each parameter θ_i, the penalty took the form $F_i(\theta_i - \theta_{A,i}^*)^2$, where F_i is the Fisher information for that parameter. This produces a total loss:

$$\mathcal{L}_{\text{total}} = \mathcal{L}_B + \lambda_{\text{EWC}} \sum_i F_i(\theta_i - \theta_{A,i}^*)^2$$

where $\mathcal{L}_B$ is the standard supervised loss on Task B and λ_{EWC} is a regularisation coefficient controlling the strength of the EWC constraint.

This approach mirrors the method introduced by [16], approximating the posterior over Task A parameters with a diagonal Gaussian centered at θ_A^*, and using the Fisher information to weight the elastic constraint according to parameter importance.

6.3 Replay

Replay mitigates forgetting by interleaving stored examples from prior tasks with training data from the current task, thereby reinforcing previously learned representations. In our implementation, we employed a simple experience replay strategy that stores Task A training data and reintroduces it during Task B learning.

At the end of Task A training, we constructed a replay buffer containing the full set of Task A training data. During Task B training, one example was sampled at random from this buffer on every training step and treated as an additional pseudo-example from Task A. The network's loss for each batch was computed as the sum of two components: the standard supervised loss on Task B input, and a loss on the replayed Task A input. Both were weighted equally, and gradients were backpropagated through the combined loss. This continual integration of prior-task information preserves performance on Task A without requiring architectural modifications. This method corresponds to experience-based rehearsal approaches in continual learning [17–19].

6.4 Modular Architecture

Modular architectures mitigate interference by allocating distinct subsets of network parameters to different tasks, preventing overlap in representational resources. In our implementation, we divided the hidden layer of a linear neural network into two non-overlapping modules, with each

task accessing only one of the two halves. Specifically, the network consisted of a shared input-to-hidden and hidden-to-output linear mapping, but with a gating mechanism that zeroed out one half of the hidden units depending on the task. By preventing shared parameter usage across tasks in this implementation, partitioning eliminates interference entirely at the cost of any transfer between tasks. This approach echoes architectural strategies in continual learning that use task-specific sub-networks to avoid forgetting [20, 21], but without additional mechanisms for leveraging prior knowledge.

The results of training networks in the Near condition (i.e. tasks that share similar but non-identical rules, with unique inputs across tasks) are shown below in (see Figure S17). We compare hidden layer geometry and behavioural outcomes—transfer, generalisation, and interference—for five network types: rich, lazy, EWC, replay, and modular.

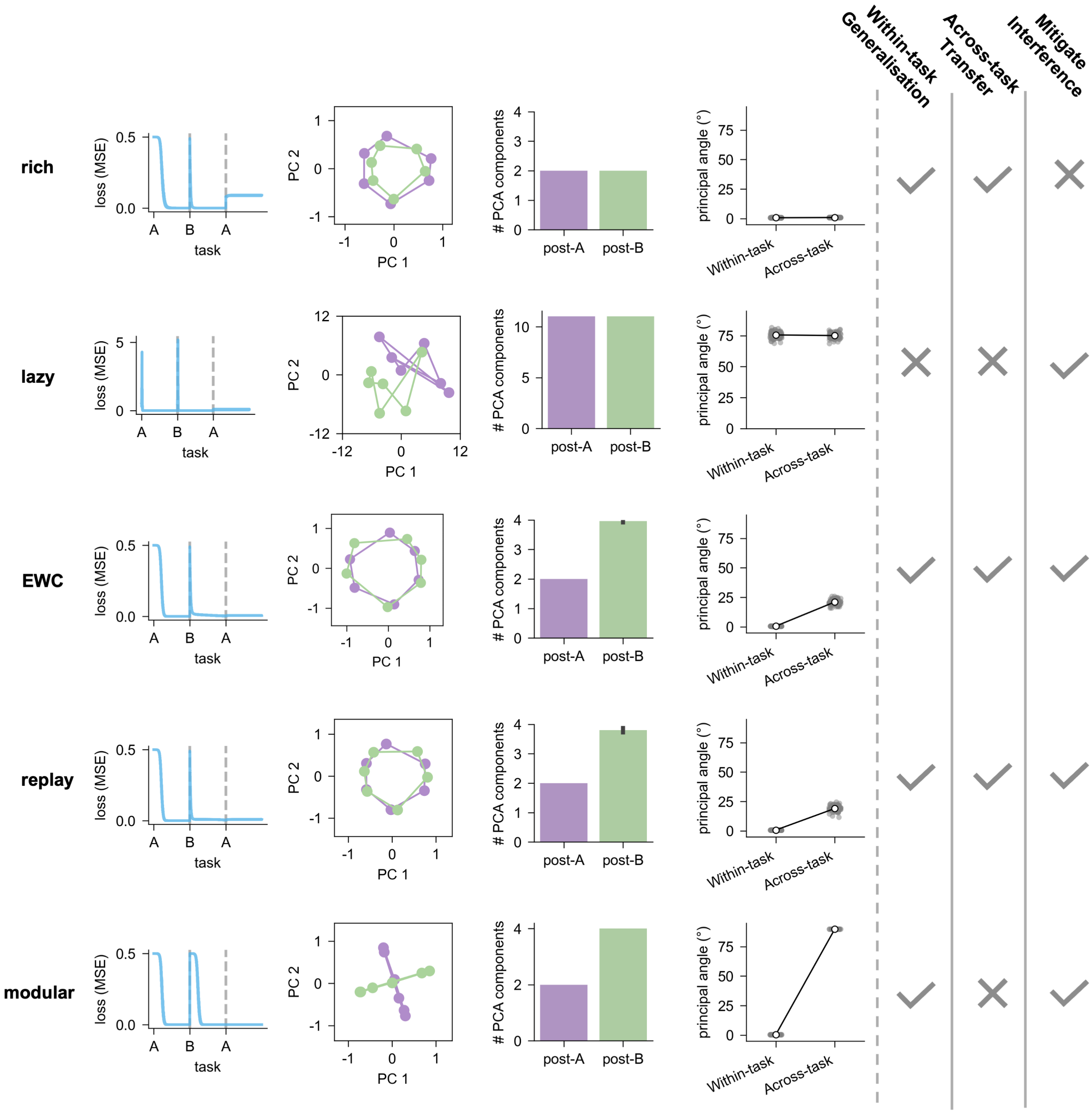

Figure S17: **Representational solutions under different model architectures.** Networks were trained on consecutive similar tasks (Near condition). Each panel illustrates how different network regimes or interference-mitigation strategies shape internal representations and behavioural outcomes in a continual learning setting: **(1)** Rich networks reuse a low-dimensional subspace across tasks, supporting strong generalisation and transfer but incurring interference. **(2)** Lazy networks develop high-dimensional, task-agnostic embeddings, reducing interference but impairing generalisation. **(3)** EWC constrains updates to parameters estimated to be important for Task A. **(4)** Replay buffers Task A training data and interleaves it during Task B training. **(5)** Modular networks isolate tasks into separate hidden units, enforcing orthogonal representations.

6.5 Comparing human profiles to model behaviours.

Given this range of behavioural profiles, we next asked: what kinds of representational regimes best match the performance profiles of human *splitters* and *lumpers*?

- **Splitters** (participants in the Near condition who avoid forgetting) show poor within-task

generalisation and low transfer, resembling lazy networks. This profile is not matched by replay, EWC, or modular solutions.

- **Lumpers** (participants in the Near condition who suffer interference) show strong generalisation and high transfer, closely resembling rich networks without interference-mitigation mechanisms.

In summary, these different methods for mitigating interference lead to distinct internal geometries associated with distinct behavioural profiles. Among them, *splitters* most closely resemble lazy networks, which rely on high-dimensional task solutions that fail to generalise to untrained data (see Figure S18).

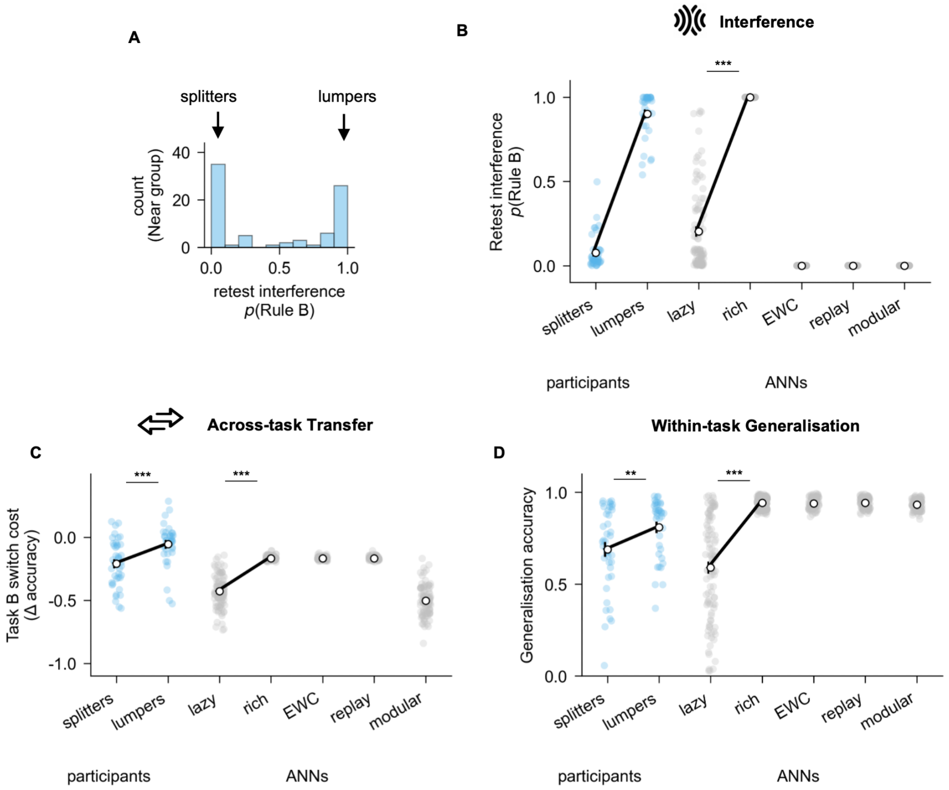

Figure S18: **Transfer, generalisation and interference behaviour under different model architectures.** This figure compares behavioural profiles of human participants ("lumpers" and "splitters") to networks trained with different interference-mitigation strategies. **(A)** Histogram of interference weights (π) used to define lumpers and splitters. **(B)** Interference across groups: splitters, lazy networks, EWC, replay, and modular networks all show reduced interference. **(C)** Transfer behaviour (change in accuracy from Task A to Task B): splitters, lazy, and modular networks all show reduced transfer. **(D)** Generalisation behaviour (accuracy for untrained stimulus in Task A): splitters and lazy networks show the lowest generalisation. Together, these results suggest that splitters most closely resemble lazy networks, while lumpers resemble rich networks.

References

[1] Kurt Hornik and Bettina Grün. movMF: An R Package for Fitting Mixtures of von Mises-Fisher Distributions. *Journal of Statistical Software*, 58:1–31, July 2014.

[2] François Raschelle. Mixture of von Mises distributions, 2020.

[3] Klaas Enno Stephan, Will D. Penny, Jean Daunizeau, Rosalyn J. Moran, and Karl J. Friston. Bayesian model selection for group studies. *NeuroImage*, 46(4):1004–1017, July 2009.

[4] L. Rigoux, K. E. Stephan, K. J. Friston, and J. Daunizeau. Bayesian model selection for group studies - revisited. *NeuroImage*, 84:971–985, January 2014.

[5] Ham Huang. Bms, 2024.

[6] Timo Flesch, Keno Juechems, Tsvetomira Dumbalska, Andrew Saxe, and Christopher Summerfield. Orthogonal representations for robust context-dependent task performance in brains and neural networks. *Neuron*, 110(7):1258–1270.e11, April 2022.

[7] Andrew M. Saxe, James L. McClelland, and Surya Ganguli. Exact solutions to the nonlinear dynamics of learning in deep linear neural networks, February 2014.

[8] Jeffrey Pennington, Samuel S. Schoenholz, and Surya Ganguli. Resurrecting the sigmoid in deep learning through dynamical isometry: Theory and practice, November 2017.

[9] Arthur Jacot, Franck Gabriel, and Clement Hongler. Neural Tangent Kernel: Convergence and Generalization in Neural Networks. In *Advances in Neural Information Processing Systems*, volume 31. Curran Associates, Inc., 2018.

[10] Lénaïc Chizat, Edouard Oyallon, and Francis Bach. On Lazy Training in Differentiable Programming. In *Advances in Neural Information Processing Systems*, volume 32. Curran Associates, Inc., 2019.

[11] Mario Geiger, Leonardo Petrini, and Matthieu Wyart. Perspective: A Phase Diagram for Deep Learning unifying Jamming, Feature Learning and Lazy Training, December 2020.

[12] Blake Woodworth, Suriya Gunasekar, Jason D. Lee, Edward Moroshko, Pedro Savarese, Itay Golan, Daniel Soudry, and Nathan Srebro. Kernel and Rich Regimes in Overparametrized Models. In *Proceedings of Thirty Third Conference on Learning Theory*, pages 3635–3673. PMLR, July 2020.

[13] Jonas Paccolat, Leonardo Petrini, Mario Geiger, Kevin Tyloo, and Matthieu Wyart. Geometric compression of invariant manifolds in neural networks. *Journal of Statistical Mechanics: Theory and Experiment*, 2021(4):044001, April 2021.

[14] Matthew Farrell, Stefano Recanatesi, and Eric Shea-Brown. From lazy to rich to exclusive task representations in neural networks and neural codes. *Current Opinion in Neurobiology*, 83:102780, December 2023.

[15] Clémentine C. J. Dominé, Nicolas Anguita, Alexandra M. Proca, Lukas Braun, Daniel Kunin, Pedro A. M. Mediano, and Andrew M. Saxe. From Lazy to Rich: Exact Learning Dynamics in Deep Linear Networks, September 2024.

[16] James Kirkpatrick, Razvan Pascanu, Neil Rabinowitz, Joel Veness, Guillaume Desjardins, Andrei A. Rusu, Kieran Milan, John Quan, Tiago Ramalho, Agnieszka Grabska-Barwinska, Demis Hassabis, Claudia Clopath, Dharshan Kumaran, and Raia Hadsell. Overcoming catastrophic forgetting in neural networks. *Proceedings of the National Academy of Sciences*, 114(13):3521–3526, March 2017.

[17] Anthony Robins. Catastrophic Forgetting, Rehearsal and Pseudorehearsal. *Connection Science*, 7(2):123–146, June 1995.

[18] David Rolnick, Arun Ahuja, Jonathan Schwarz, Timothy Lillicrap, and Gregory Wayne. Experience Replay for Continual Learning. In *Advances in Neural Information Processing Systems*, volume 32. Curran Associates, Inc., 2019.

[19] Gido M. van de Ven, Hava T. Siegelmann, and Andreas S. Tolias. Brain-inspired replay for continual learning with artificial neural networks. *Nature Communications*, 11(1):4069, August 2020.

[20] Jeongtae Lee, Jaehong Yoon, Eunho Yang, and Sung Ju Hwang. Lifelong Learning with Dynamically Expandable Networks. *CoRR*, January 2017.

[21] Andrei A. Rusu, Neil C. Rabinowitz, Guillaume Desjardins, Hubert Soyer, James Kirkpatrick, Koray Kavukcuoglu, Razvan Pascanu, and Raia Hadsell. Progressive Neural Networks, October 2022.
