# Supplementary material for: Humans and neural networks show similar patterns of transfer and interference during continual learning
Source: Nat Hum Behav. 2025 Oct 30;10(1):111–25. doi: 10.1038/s41562-025-02318-y (PMC12846915; doi:10.1038/s41562-025-02318-y)
Supplement: Supplementary file 2 — Reporting Summary [file 41562_2025_2318_MOESM2_ESM.pdf]

nature portfolio

Corresponding author(s): Eleanor Holton

Last updated by author(s): Aug 26, 2025

Reporting Summary

Nature Portfolio wishes to improve the reproducibility of the work that we publish. This form provides structure for consistency and transparency in reporting. For further information on Nature Portfolio policies, see our Editorial Policies and the Editorial Policy Checklist.

Statistics

For all statistical analyses, confirm that the following items are present in the figure legend, table legend, main text, or Methods section.

n/a	Confirmed	
☐	☒	The exact sample size (*n*) for each experimental group/condition, given as a discrete number and unit of measurement
☐	☒	A statement on whether measurements were taken from distinct samples or whether the same sample was measured repeatedly
☐	☒	The statistical test(s) used AND whether they are one- or two-sided<br>*Only common tests should be described solely by name; describe more complex techniques in the Methods section.*
☐	☒	A description of all covariates tested
☐	☒	A description of any assumptions or corrections, such as tests of normality and adjustment for multiple comparisons
☐	☒	A full description of the statistical parameters including central tendency (e.g. means) or other basic estimates (e.g. regression coefficient) AND variation (e.g. standard deviation) or associated estimates of uncertainty (e.g. confidence intervals)
☐	☒	For null hypothesis testing, the test statistic (e.g. *F*, *t*, *r*) with confidence intervals, effect sizes, degrees of freedom and *P* value noted<br>*Give P values as exact values whenever suitable.*
☒	☐	For Bayesian analysis, information on the choice of priors and Markov chain Monte Carlo settings
☒	☐	For hierarchical and complex designs, identification of the appropriate level for tests and full reporting of outcomes
☐	☒	Estimates of effect sizes (e.g. Cohen's *d*, Pearson's *r*), indicating how they were calculated

Our web collection on statistics for biologists contains articles on many of the points above.

Software and code

Policy information about availability of computer code

Data collection	Data were collected using a custom online game developed in JavaScript. Participants were recruited via Prolific.
Data analysis	All code is available on GitHub (https://github.com/eleanorholton/transfer-interference). The repository includes custom Python scripts for data analysis and simulations of artificial neural networks (ANNs). All code necessary to reproduce the results of this study is provided.

For manuscripts utilizing custom algorithms or software that are central to the research but not yet described in published literature, software must be made available to editors and reviewers. We strongly encourage code deposition in a community repository (e.g. GitHub). See the Nature Portfolio guidelines for submitting code & software for further information.

Data

Policy information about availability of data

All manuscripts must include a data availability statement. This statement should provide the following information, where applicable:

- Accession codes, unique identifiers, or web links for publicly available datasets
- A description of any restrictions on data availability
- For clinical datasets or third party data, please ensure that the statement adheres to our policy

The processed, anonymized human data generated and analyzed in this study are available on GitHub at https://github.com/eleanorholton/transfer-interference. There are no restrictions on data availability, and all relevant files are provided in CSV format. No data with mandated deposition is included in this study.

Research involving human participants, their data, or biological material

Policy information about studies with human participants or human data. See also policy information about sex, gender (identity/presentation), and sexual orientation and race, ethnicity and racism.

Reporting on sex and gender	We asked for participants' self-reported gender at the time of data collection: Out of a total of 305 participants, 157 self-reported as female. We do not include further analysis of gender, as it was not applicable to our research questions.
Reporting on race, ethnicity, or other socially relevant groupings	We did not collect data on race or ethnicity, as it was not applicable to our research questions.
Population characteristics	See section 'Research sample' in 'Behavioural & social sciences study design'
Recruitment	Participants were recruited from Prolific.co, an online platform for participant recruitment. Eligibility criteria included age (18–40 years), English language proficiency, geographical location (US or UK), a minimum approval rate of 90%, and at least 5 previous submissions on Prolific. The use of Prolific may introduce self-selection biases due to familiarity with online studies and limited generalizability to broader populations. However, as our study aims to compare learning mechanisms between humans and artificial neural networks, these biases are unlikely to influence the comparative analysis.
Ethics oversight	Ethical approval was obtained from the Oxford Central University Research Ethics Committee (Ref: R50750/RE009).

Note that full information on the approval of the study protocol must also be provided in the manuscript.

Field-specific reporting

Please select the one below that is the best fit for your research. If you are not sure, read the appropriate sections before making your selection.

☐ Life sciences ☒ Behavioural & social sciences ☐ Ecological, evolutionary & environmental sciences

For a reference copy of the document with all sections, see nature.com/documents/nr-reporting-summary-flat.pdf

Behavioural & social sciences study design

All studies must disclose on these points even when the disclosure is negative.

Study description	Data are quantitative experimental data. The study involves behavioural data collected online from a 45 minute experimental task.
Research sample	The research sample consisted of adult participants recruited via Prolific.co, an online platform frequently used for behavioural research. Participants were recruited based on the following inclusion criteria: Aged between 18 and 40 years; Native English speakers; Located in the US or the UK; Holding a minimum approval rate of 90% on Prolific; Having completed at least 5 previous studies on Prolific. A total of 202 participants were recruited for the Discovery study (151 remaining after exclusion) and 215 participants were recruited for the Replication study (154 remaining after exclusion). Of the 305 participants total remaining after exclusion, the mean age was 31.37 years (SD = 8.27) and 157 participants identified as female.
Sampling strategy	The sampling strategy was convenience sampling using Prolific.co, an online participant recruitment platform that provides a large and diverse pool of participants. Sample size for the replication study was determined based on a power analysis of the original dataset (discovery sample). The effect sizes detected in the original study suggested that a sample size of 50 participants per condition (same, near, far) would be sufficient to detect the hypothesized effects at a power level of 80%. This sample size also allows us to make direct comparisons with the original study to test the reproducibility of findings. No formal sample size calculation was conducted for the previous study.
Data collection	Data were collected using a custom-built online task coded in JavaScript, which was hosted on the lab server. Participants completed the task remotely on their own computers via Prolific.co. The task involved a visual-motor response paradigm where participants moved a dial to indicate their response on a circular display. The experimental setup was fully automated and participants completed the task independently. The experiment was designed to ensure that participants could not receive any feedback or external assistance beyond the visual feedback provided within the task. The researchers were not blind to the experimental conditions, but this was not necessary as no researchers were present during data collection. The study hypotheses were pre-registered and outlined in detail prior to data collection.
Timing	Study 1 (discovery sample) was conducted 8th-9th July 2024. Study 2 (replication sample) was conducted 21st October 2025.
Data exclusions	Data exclusions were pre-registered and applied according to the following criteria:<br><br>Tool Use Exclusion: Participants who reported using external tools (e.g., pen and paper) to remember correct responses were excluded.<br><br>Accuracy Criterion for Task A: Participants whose accuracy did not significantly exceed chance performance (defined as 90 degrees of error) during the final two blocks of training on Task A were excluded.

	Interference Analysis Exclusion: For the interference analysis, participants who did not adequately learn Task B were excluded. This was assessed by fitting responses during Task B to two von Mises distribution models representing the use of the two rules (A-rule and B-rule). Participants were excluded if the A-rule provided a better fit to their Task B responses than the B-rule.<br><br>Incomplete Data: Participants with missing or incomplete data were excluded from the analyses.<br><br>These criteria were pre-registered. A total of 51 participants were excluded from the Discovery study (202 recruited, 151 remaining) and 61 participants were excluded from the Replication study (215 recruited, 154 remaining).
Non-participation	Participants who began the task could choose to return the study on Prolific.co before completion. Approximately 16% participants returned their submission on Prolific during the study. The decision to return the study was made voluntarily by participants, and no specific reasons for return were provided by Prolific. No participants who completed the task dropped out or declined participation beyond these returns.
Randomization	Participants were assigned to one of three experimental conditions (same rule, near rule, far rule) through a process involving three separate recruitment batches on Prolific.co. Each condition was associated with a unique Prolific recruitment link that directed participants to a specific version of the task differing only in the independent variable. The description of the task on the Prolific interface was identical across conditions. Participants self-selected into one of the conditions by responding to the available recruitment link. The recruitment process was run in parallel for all three conditions. Prolific's recruitment interface ensured that participants who participated in one version of the study were not eligible to participate in any of the other conditions.

Reporting for specific materials, systems and methods

We require information from authors about some types of materials, experimental systems and methods used in many studies. Here, indicate whether each material, system or method listed is relevant to your study. If you are not sure if a list item applies to your research, read the appropriate section before selecting a response.

Materials & experimental systems

n/a	Involved in the study
☒	☐ Antibodies
☒	☐ Eukaryotic cell lines
☒	☐ Palaeontology and archaeology
☒	☐ Animals and other organisms
☒	☐ Clinical data
☒	☐ Dual use research of concern
☒	☐ Plants

Methods

n/a	Involved in the study
☒	☐ ChIP-seq
☒	☐ Flow cytometry
☒	☐ MRI-based neuroimaging

Plants

Seed stocks	NA
Novel plant genotypes	NA
Authentication	NA
